# A Multidimensional Assessment of Activities of Daily Living, Mental Status, Communication, and Social Abilities Among Older Adults in Shenzhen, China: Cross-Sectional Study

**DOI:** 10.2196/43612

**Published:** 2023-08-10

**Authors:** Jing Wang, Patrick Kwan, Gong Zhang, Mingwang Shen, Loretta Piccenna, Terence J O’Brien, Lei Zhang

**Affiliations:** 1 China-Australia Joint Research Centre for Infectious Diseases School of Public Health Xi'an Jiaotong University Health Science Centre Xi'an, Shaanxi China; 2 Department of Neuroscience The Central Clinical School Monash University Melbourne, Victoria Australia; 3 Department of Neurology Alfred Health Melbourne, Victoria Australia; 4 Departments of Medicine and Neurology The Royal Melbourne Hospital The University of Melbourne Parkville, Victoria Australia; 5 Ministry of Education Key Laboratory of Tumour Molecular Biology and Key Laboratory of Functional Protein Research of Guangdong Higher Education Institutes Institute of Life and Health Engineering, College of Life Science and Technology Jinan University Guangzhou China; 6 Key Laboratory for Disease Prevention and Control and Health Promotion of Shaanxi Province Xi'an, Shaanxi China; 7 Artificial Intelligence and Modelling in Epidemiology Program Melbourne Sexual Health Centre Alfred Health Melbourne Australia; 8 Central Clinical School Faculty of Medicine Monash University Melbourne Australia

**Keywords:** older adults, ability assessment, activities of daily living, mental status, sensory and communication, social participation

## Abstract

**Background:**

China is facing a rapidly expanding aging population. Insights into the health status of older adults are of great significance for health resource allocation and health care provision to this population.

**Objective:**

With the goal of providing a comprehensive understanding of the health status of older adults and to inform potential interventions, we investigated the level of disability and identified risk factors associated with disability among the older population (aged ≥60 years) living in China.

**Methods:**

A total of 8467 older adults living in the Chinese city of Shenzhen were enrolled in this cross-sectional study. We used a multidimensional ability assessment survey, which assessed their activities of daily living (ADL; including eating, bathing, grooming, dressing, defecation control, urination control, using a toilet unaided, transfer, flat-ground walking, stair activity), mental status (including cognitive function, aggressive behavior, depression symptoms), sensory and communication (including consciousness level, vision, hearing, communication), and social participation (including living, working, time/space orientation, distinguish persons, social communication) abilities. The impact of demographic risk factors on ability levels was analyzed using ordinal logistic regression. The correlations between the four dimensions of ability mentioned above were analyzed using Spearman correlation analysis.

**Results:**

A total of 7766 participants were effectively assessed. The participants’ average age was 70.64 (SD 8.46) years comprising 56.53% females. The overall ability level was classified as mildly, moderately, and severely impaired for 27.57% (n=2141), 2.83% (n=220), and 4.28% (n=332) of the 7766 participants, respectively. With increasing age, the proportion of impaired participants increased from 17.62% (365/2071) in the age group 60-64 years to 91.3% (253/277) in the age group above 90 years (*P*<.001), corresponding to an approximate 10% rise for every 5-year age increment. The odds of having more severe overall ability impairment in females was 1.15 times that in males (odds ratio [OR] 1.15, 95% CI 1.04-1.28). Participants who were divorced or widowed had a higher risk of more severe overall ability impairment than those currently married (OR 1.98, 95% CI 1.68-2.33). Participants living with nonrelatives had an increased risk of more severe overall ability impairment than those living alone (OR 2.38, 95% CI 1.46-3.91). Higher education level was a protective factor of overall ability impairment (college degree or above: OR 0.32, 95% CI 0.24-0.42). The four dimensions of ability assessed were significantly correlated; a low score for ADL was significantly correlated with poorer mental status, sensory and communication, and social participation (all *P*<.001).

**Conclusions:**

The proportion of disability among Chinese older adults increases with age, being female, having lower education levels, being divorced or widowed, and living with nonrelatives. Impairment in ADL ability is significantly correlated with poor mental status, social participation, and sensory and communication abilities. A holistic approach to improving the health of the older population is recommended in China.

## Introduction

Population aging is accelerating worldwide due to declining birth rates, economic development, and improved medical and health services that extend human life longevity [1]. It is estimated that the world’s population aged 60 years and over will rise by 56% from 901 million to 1.4 billion during 2015-2030 [2]. In China, an estimated 176 million individuals were aged 65 years or above in 2019, accounting for 12.6% of the Chinese population. This indicates that China has entered a fast track of population aging [3]. By 2050, the older population aged 65 years or above in China is expected to reach 330 million, accounting for more than 20% of the world’s older population [4]. Notably, China is currently in a stage of rapid development and the necessary resources required to address the aging population, including financial, material, and human resources, have not been fully prepared [5]. The rapidly aging population has brought substantial challenges to China’s health care system, in particular with respect to the allocation of limited health resources and pension services required to secure a healthy life in older age [6].

Aging is accompanied by multiple chronic diseases such as hypertension, diabetes, cardiovascular diseases, and dementia. These conditions are often due to chronic, low-grade inflammation; macromolecular and organelle dysfunction; stem cell and progenitor changes; and cellular senescence [7,8]. According to the Fourth National Health Data Survey in China, the prevalence of chronic diseases was 64.5% in people aged above 65 years [9]. Moreover, mental health has also become a prevalent issue among the older Chinese population. Li et al [10] reported a depression prevalence of 11.6% among community-dwelling older adults and of 18.1% among older hospital inpatients in China. Physical and mental disabilities are known to have a profound impact on the quality of life experienced by older adults. This may be attributed to the challenges faced by this population in creating wealth due to their disabilities, which often place them in a lower socioeconomic profile rendering them highly susceptible to social isolation [11]. China has the largest population of disabled older adults in the world [12]: approximately 41 million older people were considered disabled and semidisabled in 2015, accounting for approximately 18.3% of people aged above 60 years in the country [13]. This number is expected to increase to 62 million in 2030 and to 98 million in 2050 [14]. In this context, to improve the quality of life of older people, it is important to adopt personalized multicomponent prevention and intervention measures to improve or maintain their overall abilities [15]. Hence, multidimensional ability assessments for older adults would provide the necessary evidence to inform prevention and intervention strategies.

To date, numerous studies have been conducted to assess the multidimensional abilities of older adults in developed countries with comprehensive assessment tools used in community settings, such as the Resident Assessment Instrument for Home Care and the EASYcare instrument, which have been widely used to evaluate physical functioning, including activities of daily living (ADL), mental functioning, and social functioning [16,17]. In comparison, there have been limited studies related to multidimensional ability assessment in the older population in China. Previous studies on ability assessment in older adults mainly focused on the ability to fulfill ADL [18,19]. Yet, as mentioned, a more comprehensive approach that assesses various aspects, including physical, mental, and social function, among the older population would provide more holistic insights into this population’s overall health status [20-24]. In addition, previous studies in China have reported that the overall ability of the older population was largely related to their sex, education, and living condition; however, the findings were not consistent across different settings [25,26].

In this study, we investigated a large group of older adults in the Chinese city of Shenzhen. We aimed to comprehensively assess the older population’s overall ability across multiple dimensions, including ADL, mental status, sensory and communication, and social participation. We further explored the potential demographic factors associated with the impairment of overall ability and the correlation between the four dimensions of ability. The study findings will provide evidence to inform interventions, health policy, and health resources allocation for the older population in a Chinese setting.

## Methods

### Setting

The study was conducted across two districts (57 community centers) in the city of Shenzhen, China. The rollout of the survey and data collection was coordinated by a community-based nursing home privately founded in 2016. The nursing home provides aged care, nursing services, rehabilitation training, and mass health education for older adults living in the community. The nursing home has employed a smart information cloud platform that can generate an electronic version of the questionnaire used to assess the overall ability of the older population. The information system enabled a sanity check on the input information and reported errors if the input information is inappropriate, providing a certain accuracy guarantee for the data. Data were securely backed up and stored on the cloud platform.

### Study Design

A cross-sectional study design was used and the study was conducted according to STROBE (Strengthening the Reporting of Observational Studies in Epidemiology) guidelines (see Multimedia Appendix 1). From April 2017 to December 2019, a total of 8467 older adults aged ≥60 years and owning a local household registration were recruited through the nursing home mentioned above. The nursing home’s catchment area covers a total of 57 local communities and 8467 older adults. Of the 8467 recruited older adults, 7965 completed assessments that were ultimately collected, excluding those who were absent at the time of the assessment or chose not to participate. During the assessment, the participants were routinely asked to provide demographic information and complete a standardized ability assessment survey. The survey was conducted using the “Ability Assessment for Older Adults” questionnaire, which is the standardized questionnaire published by the Ministry of Civil Affairs of the People’s Republic of China. This questionnaire was jointly drafted by members of the China Social Welfare Association, School of Nursing of Peking University, China Medical Women’s Association, and other units regarding the existing tools for the ability assessment of older adults in China and abroad [27]. A team of 30-50 staff members from the nursing home was trained to conduct face-to-face interviews to administer the survey. After they explained the purpose and significance of the survey, the participants provided informed consent. For the participants who were unable to complete the survey independently due to physical or cognitive disabilities, family members or primary caregivers answered the survey on their behalf. The collected data were then entered into the smart information cloud platform to inform the future provision of services. In this study, we retrospectively examined the information provided by the participants at enrollment.

### Inclusion and Exclusion Criteria

The inclusion criteria for participants were as follows: (1) aged 60 years or older, (2) have a local household registration and have lived there for more than 6 months, (3) willing to participate in the survey for ability assessment, and (4) completed the demographic information collection form and the overall ability assessment questionnaire. Note that criteria 1-3 were utilized for selecting participants during the field survey, whereas the fourth criterion was employed during the data analysis phase.

Participants were excluded if there was missing information on the (1) variable “accidents over the last 30 days” in the demographic information collection form, (2) ADL assessment part of the overall ability assessment questionnaire, (3) mental status assessment part of the overall ability assessment questionnaire, (4) sensory and communication assessment part of the overall ability assessment questionnaire, and (5) social participation assessment part of the overall ability assessment questionnaire. All five criteria were used for excluding participants during the data analysis phase.

### Ethics Approval

Ethics approval was obtained from the Biomedical Ethics Committee of the Medical Department of Xi’an Jiaotong University (Human Ethics Approval Number 2020-10). The data collection process was carried out using a deidentified approach to ensure the confidentiality of the study participants. During the study period, participants were offered certain services, including meal delivery and room cleaning, by the nursing home mentioned above, without any form of compensation. Prior to participation in the study, all participants provided their oral informed consent, in line with established ethical guidelines and regulatory requirements governing human research studies. This crucial step ensured that participants were fully informed of the study’s objectives, procedures, and potential risks, and made an informed decision to voluntarily participate in the study.

### Data Collection

We collected demographic and ability assessment data from the information service platform. To fulfill one of our research objectives, we selected demographic variables that were commonly collected from all study participants and had been previously shown to contribute to the ability level of older adults [25,26]. Ultimately, we selected five variables (age, sex, education, marital status, and living situation) as the demographic variables of interest for this analysis.

The participants’ overall ability assessment consisted of four main dimensions. First, the ADL assessment included 10 daily activity items (eating, bathing, grooming, dressing, defecation control, urination control, using a toilet unaided, transfer, flat-ground walking, stair activity) and had a total score of 100 points (see Multimedia Appendix 2 for details). The score was graded according to four levels (100 points, unimpaired; 65-95 points, mildly impaired; 45-60 points, moderately impaired; ≤40 points, severely impaired). Second, the mental status assessment included three items (cognitive function, aggressive behavior, depression symptoms) with 6 points in total, which were divided into four levels (0 points, unimpaired; 1 point, mildly impaired; 2-3 points, moderately impaired; 4-6 points, severely impaired). Third, the sensory and communication assessment included four items (consciousness level, vision, hearing, and communication). The assessment results were classified according to certain conditions and were divided into four levels (unimpaired, mildly impaired, moderately impaired, and severely impaired). Fourth, the social participation assessment included five items (living ability, working ability, time/space orientation, distinguish persons, social communication ability) with a total score of 20 points divided into four levels (0-2 points, unimpaired; 3-7 points, mildly impaired; 8-13 points, moderately impaired; 14-20 points, severely impaired).

The classification of the overall ability level was predetermined by the survey according to the standard of the Chinese Ministry of Civil Affairs [27]. In brief, if all ADL, mental status, and sensory and communication dimensions were classified as “unimpaired” and only social participation was classified as “unimpaired” or “mildly impaired,” then the overall ability result was classified as “unimpaired.” The overall ability level was defined as “mildly impaired” if it satisfied one of the following: (1) the result of ADL was “unimpaired” but at least one of mental status or sensory and communication was classified as “mildly impaired” or above or the social participation was classified as “moderately impaired”; (2) the ADL was classified as “mildly impaired” but at least one of the other three dimensions was classified as “unimpaired” or “mildly impaired.” The overall ability was classified as “moderately impaired” if it satisfied one of the following: (1) the result of ADL was “mildly impaired” but the other three dimensions were all classified as “moderately impaired” or at least one dimension was classified as “severely impaired”; (2) the result of ADL was “moderately impaired” and one or two of the other three dimensions were classified as “mildly impaired” or “moderately impaired.” The overall ability was defined as “severely impaired” if it satisfied one of the following: (1) the result of ADL was “severely impaired,” (2) the results of four dimensions were all “moderately impaired,” and (3) the result of ADL was “moderately impaired” and at least one of the other three dimensions was classified as “severely impaired.” Notably, participants with cognitive impairment/dementia or mental illness would raise the original level by one level. Participants with two or more falls, choking, suicide, and missing in the past 30 days would raise the original level by one level. Participants in a coma would be assessed as “severely impaired.”

### Statistical Analysis

Data management and statistical analysis were completed using Microsoft Excel 2016 and R 4.1.1; statistical significance was set at *P*<.05. Continuous variables are described by mean and SD, whereas categorical variables are described by frequency (n) and percentage (%). Data between different groups were compared with the χ^2^ or Fisher test for categorical data and then the proportion test was used for posthoc multiple comparisons; *P* values were adjusted using Bonferroni correction. Ordinal logistic regression was used to analyze the association between demographic characteristics and the severity of the overall ability impairment. In this model, the assessment results of the overall ability of participants were used as the dependent variable and the demographic variables that exhibited statistical significance based on the χ^2^ or Fisher test were used as independent variables. The η coefficient, SE, and *t* value were estimated to determine the statistical significance of each independent variable in the model. Associations between the severity of the overall ability impairment and demographic characteristics were also assessed by odds ratios and corresponding 95% CIs. The correlation between the various assessment items was analyzed using Spearman correlation analysis.

## Results

### Demographic Characteristics

**Figure 1:**
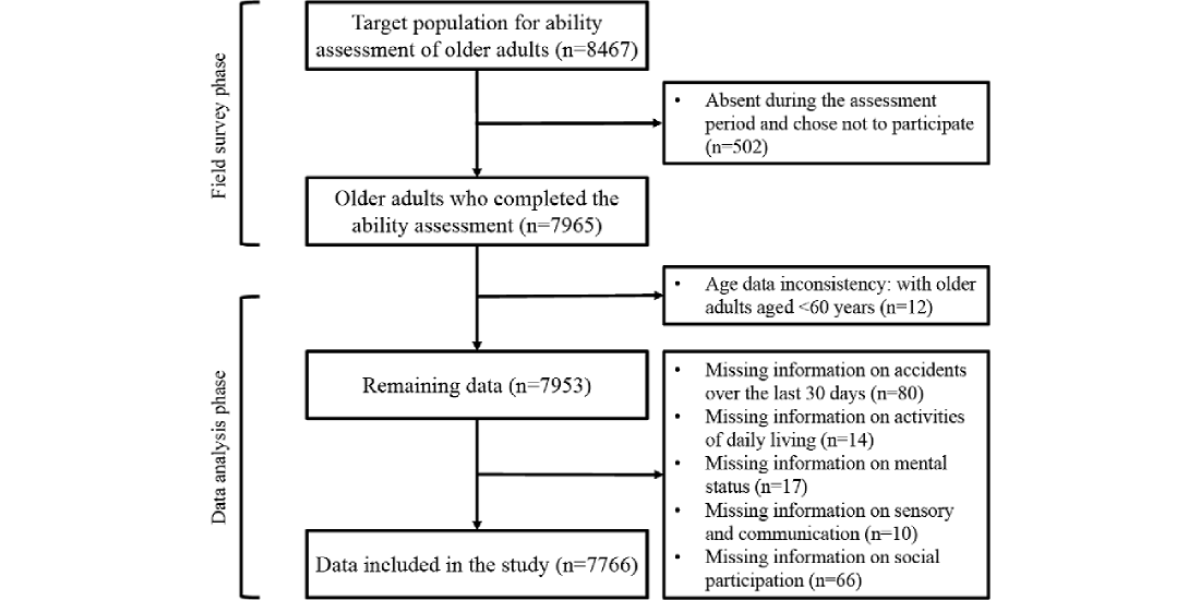
Flow chart for the selection of participants.

According to the inclusion and exclusion criteria, 7766 participants out of 8467 individuals were selected for the analysis (Figure 1). Among the 7766 participants, the average age was 70.64 (SD 8.46) years, ranging from 60 to 109 years (Table 1). There were 3376 (43.47%) males and 4390 (56.53%) females. Among the 7766 participants, 45.58% (n=3540) had primary or junior high school education levels and 13.67% (n=1062) were divorced or widowed. Most participants (3745/7766, 48.22%) were living with their children before enrollment.

**Table 1:** Demographic characteristics of study participants according to ability impairment based on the Ability Assessment for Older Adults questionnaire (N=7766).

Variable		Responses, n (%)	Overall ability level, n (%)					Pearson χ^2^ or Fisher exact test			*P* value	
			Unimpaired (n=5073, 65.32%)	Mildly impaired (n=2141, 27.57%)	Moderately impaired (n=220, 2.83%)	Severely impaired (n=332, 4.28%)						
**Age group (years)**									1875.61			<.001
	60-64	2071 (26.67)	1706 (82.38)	327 (15.79)	22 (1.06)	16 (0.77)						
	65-69	2267 (29.19)	1705 (75.21)	493 (21.75)	33 (1.45)	36 (1.59)						
	70-74	1400 (18.03)	935 (66.79)	406 (29.00)	29 (2.07)	30 (2.14)						
	75-79	720 (9.27)	395 (54.86)	272 (37.78)	21 (2.92)	32 (4.44)						
	80-84	597 (7.69)	210 (35.18)	287 (48.07)	35 (5.86)	65 (10.89)						
	85-89	434 (5.59)	98 (22.58)	228 (52.53)	40 (9.22)	68 (15.67)						
	≥90	277 (3.56)	24 (8.66)	128 (46.21)	40 (14.44)	85 (30.69)						
**Sex**									91.10			<.001
	Male	3376 (43.47)	2403 (71.18)	782 (23.16)	75 (2.22)	116 (3.44)						
	Female	4390 (56.53)	2670 (60.82)	1359 (30.96)	145 (3.30)	216 (4.92)						
**Education**									716.27			<.001
	Illiterate/semi-illiterate	500 (6.44)	120 (24.00)	247 (49.40)	45 (9.00)	88 (17.60)						
	Primary or junior high school	3540 (45.58)	2268 (64.07)	1027 (29.01)	116 (3.28)	129 (3.64)						
	Senior high or technical school	1436 (18.49)	1139 (79.32)	246 (17.13)	17 (1.18)	34 (2.37)						
	College degree or above	633 (8.15)	516 (81.52)	100 (15.80)	7 (1.10)	10 (1.58)						
	Missing	1657 (21.34)	1030 (62.16)	521 (31.44)	35 (2.11)	71 (4.29)						
**Marital status**									641.38			<.001
	Never married	23 (0.30)	10 (43.48)	6 (26.09)	4 (17.39)	3 (13.04)						
	Currently married	5098 (65.65)	3694 (72.46)	1186 (23.26)	101 (1.98)	117 (2.30)						
	Divorced or widowed	1062 (13.67)	403 (37.95)	442 (41.62)	79 (7.44)	138 (12.99)						
	Missing	1583 (20.38)	966 (61.02)	507 (32.03)	36 (2.27)	74 (4.68)						
**Living situation**									394.89			<.001
	Live alone	360 (4.64)	139 (38.61)	168 (46.67)	21 (5.83)	32 (8.89)						
	Live with spouse	2342 (30.16)	1611 (68.79)	628 (26.82)	46 (1.96)	57 (2.43)						
	Live with children	3745 (48.22)	2611 (69.72)	883 (23.58)	106 (2.83)	145 (3.87)						
	Live with other relatives	64 (0.82)	35 (54.69)	21 (32.81)	4 (6.25)	4 (6.25)						
	Live with nonrelatives	73 (0.94)	6 (8.22)	30 (41.09)	11 (15.07)	26 (35.62)						
	Nursing home	16 (0.21)	4 (25.00)	4 (25.00)	4 (25.00)	4 (25.00)						
	Other means and missing	1166 (15.01)	667 (57.20)	407 (34.91)	28 (2.40)	64 (5.49)						

### Ability Assessment

In brief, 65.32% of participants’ overall ability was assessed as unimpaired, 27.57% as mildly impaired, 2.83% as moderately impaired, and 4.28% as severely impaired (Table 1). For ADL, 81.97% of participants were classified as unimpaired, whereas 13.80%, 1.47%, and 2.76% were classified as mildly, moderately, and severely impaired, respectively. For social participation, 85.63% of participants were classified as unimpaired, whereas 8.63%, 3.23%, and 2.51% were classified as mildly, moderately, and severely impaired, respectively. For sensory and communication, 81.73% of participants were classified as unimpaired, whereas 11.79%, 5.02%, and 1.46% were classified as mildly, moderately, and severely impaired, respectively. For mental status, 80.03% of participants were classified as unimpaired, whereas 15.75%, 3.78%, and 0.44% were classified as mildly, moderately, and severely impaired, respectively.

### Impact of Demographic Characteristics on Overall Ability

**Figure 2:**
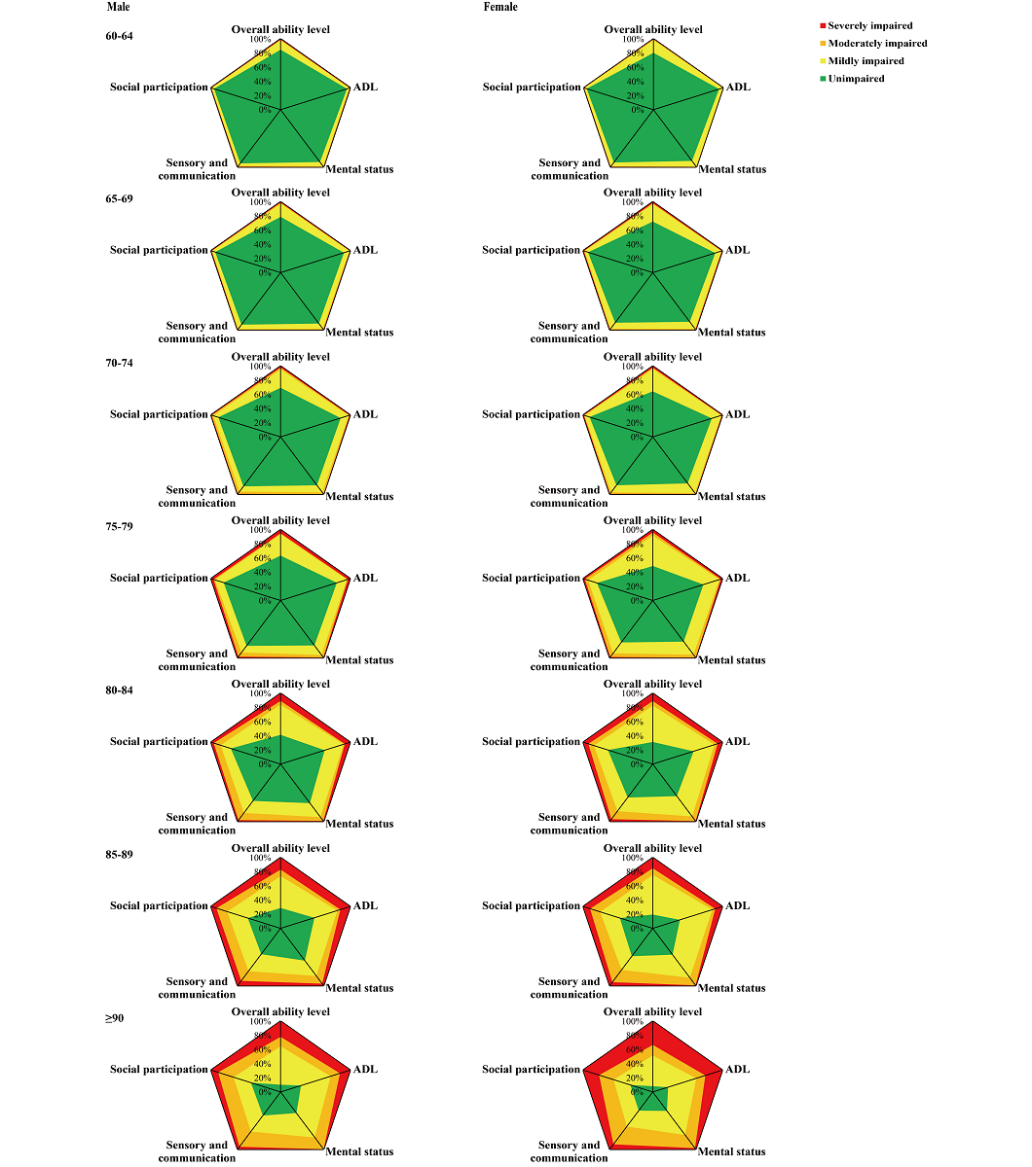
Radar maps showing the distribution of various impaired levels of ability dimensions stratified by sex and age groups. ADL: activities of daily living.

The association of ability levels with demographic characteristics is summarized in Table 1; detailed results of pairwise comparisons are presented in Multimedia Appendix 3. Age was a key factor that influenced the overall ability of the participants (Table 1, Figure 2). With increasing age, the proportion of impaired participants gradually increased from 17.62% (365/2071) in the age group 60-64 years to 91.34% (253/277) in the age group above 90 years (*P*<.001), corresponding to an approximately 10% rise for every 5-year age increment. In particular, the respective proportion of moderately and severely impaired participants increased from 1.06% and 0.77% in the age group 60-64 years to 14.44% and 30.69% in the age group above 90 years (Table 1). Further, the proportion of overall ability impairment was significantly higher in female participants than in male participants (39.18% vs 28.82%, *P*<.001). The proportion of overall ability impairment was much higher in illiterate or semi-illiterate participants (76.00%) than in those with a college degree or above (18.48%, *P*<.001). The proportion of overall ability impairment was higher in divorced or widowed (62.05%) or never-married (56.52%) participants than in those currently married (27.54%, *P*<.001 and *P*=.02 respectively). A higher proportion of participants who lived alone (61.39%) had an ability impairment compared with those who lived with their spouse (31.21%) or children (30.28%) (all *P*<.001).

### Ordinal Logistic Regression Analysis for the Severity of Overall Ability Impairment

The analysis results revealed that age, sex, education, marital status, and living situation were significantly associated with the severity of the overall ability impairment (Table 2). Specifically, the odds of having more severe overall ability impairment increased with age; for participants aged above 90 years, the odds of having more severe overall ability impairment was 23.92 times that of participants aged 60-64 years. The odds of having more severe overall ability impairment in females was 1.15 times that in males. Participants who were divorced or widowed were associated with a higher risk of more severe overall ability impairment than those currently married. In addition, participants living with nonrelatives had an increased risk of more severe overall ability impairment than those living alone. In contrast, higher education levels (college degree or above) emerged as a protective factor of the overall ability impairment.

**Table 2:** Ordinal logistic regression analysis of demographic characteristics associated with the severity of the overall ability impairment.

Variable			η coefficient (SE)		*t* value		*P* value		OR^a^ (95% CI)
**Age group (years)**									
	60-64 (reference)	N/A^b^		N/A		N/A		1.00	
	65-69	0.40 (0.08)		5.23		<.001		1.49 (1.28-1.73)	
	70-74	0.76 (0.08)		9.22		<.001		2.13 (1.82-2.51)	
	75-79	1.19 (0.10)		12.49		<.001		3.30 (2.74-3.98)	
	80-84	1.92 (0.10)		18.93		<.001		6.81 (5.59-8.31)	
	85-89	2.30 (0.12)		19.69		<.001		9.94 (7.91-12.50)	
	≥90	3.17 (0.14)		22.65		<.001		23.92 (18.18-31.50)	
**Sex**									
	Male (reference)	N/A		N/A		N/A		1.00	
	Female	0.14 (0.05)		2.64		.01		1.15 (1.04-1.28)	
**Education**									
	Illiterate or semi-illiterate (reference)	N/A		N/A		N/A		1.00	
	Primary or junior high school	–0.40 (0.10)		–3.88		<.001		0.67 (0.55-0.82)	
	Senior high or technical school	–0.91 (0.12)		–7.57		<.001		0.40 (0.32-0.51)	
	College degree or above	–1.15 (0.15)		–7.84		<.001		0.32 (0.24-0.42)	
	Missing	–0.83 (0.17)		–4.96		<.001		0.43 (0.31-0.60)	
**Marital status**									
	Never married	1.28 (0.44)		2.93		<.001		3.60 (1.51-8.45)	
	Currently married (reference)	N/A		N/A		N/A		1.00	
	Divorced or widowed	0.68 (0.08)		8.26		<.001		1.98 (1.68-2.33)	
	Missing	0.34 (0.15)		2.36		.02		1.41 (1.06-1.88)	
**Living situation**									
	Live alone (reference)	N/A		N/A		N/A		1.00	
	Live with spouse	0.15 (0.13)		1.17		.24		1.16 (0.91-1.49)	
	Live with children	–0.18 (0.12)		–1.52		.13		0.84 (0.67-1.05)	
	Live with other relatives	–0.09 (0.29)		–0.30		.77		0.92 (0.51-1.61)	
	Live with nonrelatives	0.87 (0.25)		3.46		<.001		2.38 (1.46-3.91)	
	Nursing home	1.20 (0.47)		2.58		.01		3.33 (1.32-8.31)	
	Other means and missing	0.24 (0.14)		1.77		.08		1.27 (0.98-1.66)	

^a^OR: odds ratio.

^b^N/A: not applicable.

### Correlations of Ability Assessment Scores With Various Assessment Dimensions

**Figure 3:**
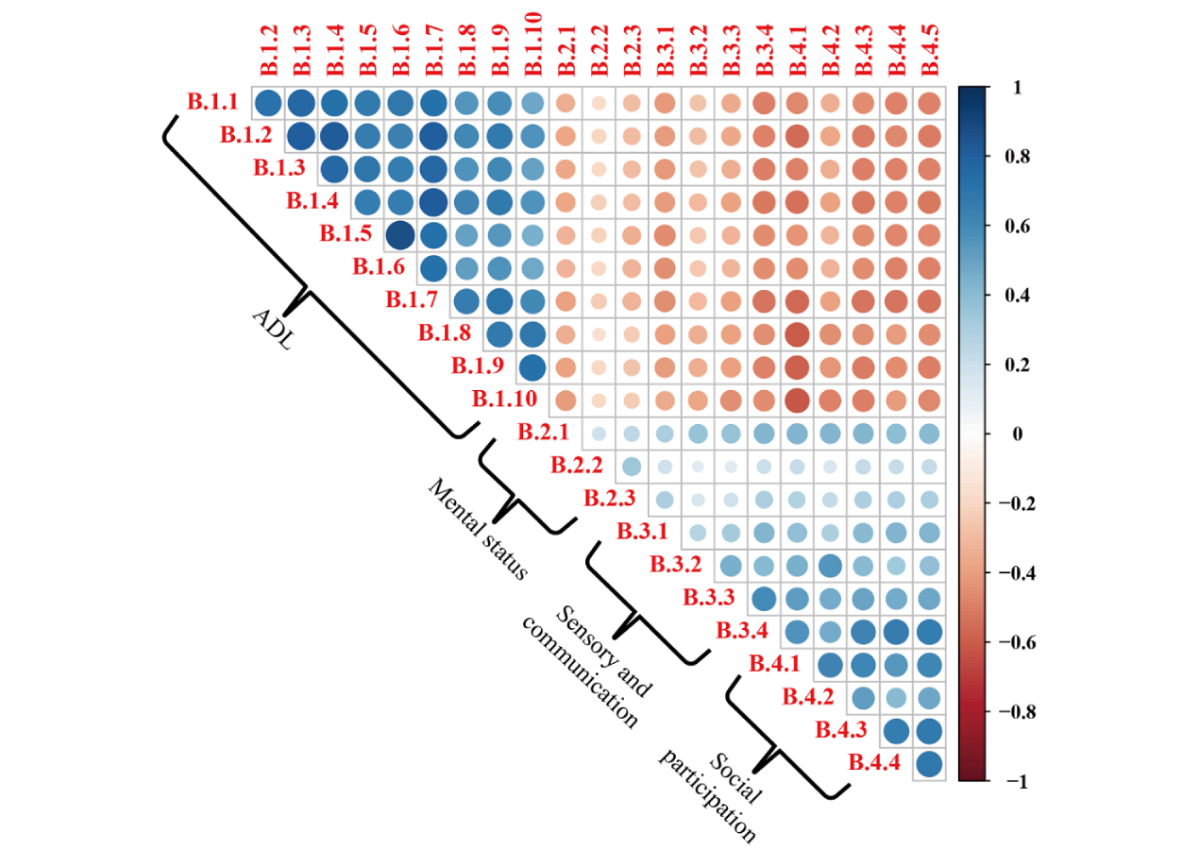
Correlation between the scores of individual items in the ability assessment. ADL: activities of daily living. See Multimedia Appendix 2 for the specific questionnaire items.

Figure 3 demonstrates that the four assessment dimensions were significantly correlated. Items within each dimension were positively correlated with other items in the same dimension (all *P*<.001). Overall, poor ADL was significantly correlated with poor mental status, sensory and communication, and social participation (all *P*<.001).

## Discussion

### Principal Findings

Our study assessed the health status of a large older population in the Chinese city of Shenzhen. Since 95% of Shenzhen citizens migrated from other places all over China, our study provides certain generality of the Chinese population. We found that nearly 34.7% of participants’ overall ability was impaired, which is higher than the reported older adult disability rate of 28.5% (25.9%-31.2%) in a recently published meta-analysis of other relevant studies from the Chinese population [28]. The proportion of moderately and severely impaired participants (7.11%) is also slightly higher than the findings from the Sixth Population Census of China (2.95%) in 2010 [29]. The differences in population size, the definition of disability, and the time of the investigation may all contribute to the difference in this result. We found that older age, being female, having lower education levels, being divorced or widowed, and living with nonrelatives were important factors associated with ability impairment. In particular, with every 5-year increment in age, the proportion of ability impairment increased by approximately 10%. Our study found that the four dimensions of ADL, mental status, sensory and communication, and social participation were significantly correlated. Poor ability in one dimension also indicated poor abilities in the other three dimensions.

Our study demonstrated that age was a strong risk factor for overall ability impairment in the Chinese community-dwelling older population and its impact increased with age. Our finding is consistent with a previous study indicating that the disability rate in older adults doubles with every 5-year age increment [30]. With aging, physical function deteriorates gradually, and nutrient intake and assimilation may be deficient and imbalanced. The prevalence of multimorbidity increases substantially with age; the proportion of adults over the age of 65 years and 85 years with multimorbidity is more than 60% and 80%, respectively [31]. Multimorbidity such as hypertension, diabetes, and cardiovascular disease can considerably reduce ability levels during a late-life stage [32,33]. Further, the risk of neurodegenerative diseases such as Alzheimer disease and Parkinson disease also increases with age, leading to cognitive dysfunction and a reduced ability level.

Our study found a significant 15% higher risk of more severe overall ability impairment in females than in males. This is in agreement with previous findings [25,26]. A similar sex difference has also been documented in various income-developed countries, including the United States and Korea, and in developing countries such as Mexico and Indonesia [34]. The sex difference may be due to two reasons. First, females generally have a longer life expectancy than males [35]. At the same time, since males are more likely to suffer from common fatal diseases, the number of disability cases in males is lower than that for females [36]. Second, major diseases that lead to disability, such as arthritis and depression, are more common in females [37].

Our finding that a lower education level was associated with a more severe overall ability impairment risk also echoes previous studies [38,39]. Several factors may contribute to this association. First, older adults with a higher education level may have more opportunities in engaging in employment after retirement, leading to a wider social network and better social support [40]. Second, a higher education level also contributes to a better socioeconomic status, giving older adults access to wider and better health care when suffering from diseases [41]. Third, older adults with a higher education level usually have a greater awareness of self-care and self-protection, which results in a healthier lifestyle and lower disability risk.

We identified that older adults who were divorced or widowed were more likely to have severe overall ability impairment compared with those currently married as the reference condition. This finding also echoes the findings of other researchers [42,43]. Marriage is a core relationship for Chinese adults, and in this setting, remaining married in general implies greater satisfaction in the social relationship and well-being of older adults [44]. Further, Chinese couples in stable marriages are often able to accumulate more wealth to secure better health care and medical services than others [45]. 

Our study found that four dimensions of the ability assessment were highly correlated. Consistently, previous studies also found that ADL ability had a significant impact on cognitive function [46,47] and that more social participation helped maintain the instrumental daily living ability of older adults [48-50]. Our research indicates the need for a comprehensive approach to care for older adults, supporting their physical, mental, and social well-being. By administering the questionnaire employed in this study to assess older adults’ abilities across these dimensions, we can determine and follow their individual status and identify aspects that should be improved, allowing for tailored interventions. To this end, smart devices are becoming a viable option to support the health and independence of the older population. These devices have become increasingly prevalent with rapid advancements in photoelectronics and information technology, which can assist with daily activities, promote mental acuity, enhance sensory function, and facilitate social engagement [51].

The main contribution of our study lies in the use of a more comprehensive assessment tool to assess the ability of older adults. With the increase in aging, there has been a growing emphasis on healthy aging. Achieving healthy aging entails ensuring that older adults are healthy in multiple dimensions, including physical, mental, and social participation [52]. Therefore, as previous studies in China have assessed ability in only one dimension, they failed to provide a comprehensive reflection of the overall ability of older adults. Our study reinforces the importance of a multidimensional assessment of older adults’ ability as a means to develop comprehensive interventions that support healthy aging. Our study provides important evidence to inform the government and health providers about the health profile of community-dwelling older adults, and serves as a valuable reference for the development of services and resource allocation for the older population.

### Limitations and Future Implications

Our study has several limitations. First, the data collected were mainly from one district in a southern Chinese city and hence may not be representative of other Chinese settings with different climates. Second, although the sample size of this study was large, there were relatively fewer participants in categories with certain demographic characteristics, such as the number of never-married (single) participants with moderately impaired overall ability. Third, due to missing data on other potential confounders such as disease and lifestyle factors, we were unable to include them in our study. This may affect our results; for instance, disease complications are more likely to affect the ability of older adults at a higher age and therefore the effect of age on the ability assessment may have been overestimated in our study. Fourth, our study followed a cross-sectional design; therefore, evidence for causal associations will need to be further investigated. Nevertheless, our multidimensional assessment has provided valuable evidence to inform the health status of the older population. As a part of future investigations, we plan to integrate these community-based data with hospital-based electronic medical records. This integration may form a much larger database and enable us to investigate the complex factors that affect the health of the older population in greater detail to provide additional evidence to support healthy aging initiatives.

### Conclusions

In conclusion, this study found a high level of disability among the older population living in Shenzhen, China. The proportion of disability increased with age, being female, having lower education levels, being divorced or widowed, and living with nonrelatives. Impairment in ADL ability was significantly correlated with poor mental status, social participation, and sensory and communication ability. Holistic interventions targeting these factors should be implemented to prevent or delay the development of disability in the Chinese older population.

## Appendix

Multimedia Appendix 1 STROBE 2007 (v4) Statement—Checklist of items that should be included in reports of cross-sectional studies.

Multimedia Appendix 2 English translation of the Ability Assessment for Older Adults questionnaire.

Multimedia Appendix 3 *P* values for pairwise differences in the proportion of overall ability impairment between different demographic groups.

## Abbreviations

ADL: activities of daily living
STROBE: Strengthening the Reporting of Observational Studies in Epidemiology

## Data Availability

The datasets used and analyzed during the current study are available from the corresponding author on reasonable request.
