# Supplementary material for: A Multidimensional Assessment of Activities of Daily Living, Mental Status, Communication, and Social Abilities Among Older Adults in Shenzhen, China: Cross-Sectional Study
Source: JMIR Public Health Surveill. 2023 Aug 10;9:e43612. doi: 10.2196/43612 (PMC10450528; doi:10.2196/43612)
Supplement: Multimedia Appendix 2 [file publichealth_v9i1e43612_app2.doc]

**THE TRANSLATION OF QUESTIONNAIRE**

**Ability Assessment for Older Adults**

**B.1 Assessment of activities of daily living**

| **B.1.1**  **Eating** | □ | 10 points: Can eat independently in a reasonable time |
| --- | --- | --- |
|  |  | 5 points: Need help partly |
|  |  | 0 points: Need great help or rely entirely on others |
| **B.1.2**  **Bathing** | □ | 5 points: Can complete the bathing process independently after preparing for the bathwater |
|  |  | 0 points: Need help from others in the bathing process |
| **B.1.3**  **Grooming** | □ | 5 points: Can complete independently |
|  |  | 0 points: Need help from others |
| **B.1.4**  **Dressing** | □ | 10 points: Can complete independently |
|  |  | 5 points: Need help partly |
|  |  | 0 points: Need great help or rely entirely on others |
| **B.1.5**  **Defecation control** | □ | 10 points: Can control |
|  |  | 5 points: Occasionally out of control ( < 1 time per week ), or need to be prompted by others |
|  |  | 0 points: Out of control completely |
| **B.1.6**  **Urination control** | □ | 10 points: Can control |
|  |  | 5 points: Occasionally out of control (< 1 time per day but > 1 time per week), or need to be prompted by others |
|  |  | 0 points: Out of control completely |
| **B.1.7**  **Using a toilet unaided** | □ | 10 points: Can complete independently |
|  |  | 5 points: Need help partly |
|  |  | 0 points: Need great help or rely entirely on others |
| **B.1.8**  **Transfer** | □ | 15 points: Can complete independently |
|  |  | 10 points: Need help partly |
|  |  | 5 points: Need great help |
|  |  | 0 points: Rely entirely on others |
| **B.1.9**  **Flat-ground walking** | □ | 15 points: Can walk 45 meters on the flat independently |
|  |  | 10 points: Need help partly |
|  |  | 5 points: Need great help |
|  |  | 0 points: Rely entirely on others |
| **B.1.10**  **Stair activity** | □ | 10 points: Can complete independently (10-15 steps up and down continuously) |
|  |  | 5 points: Need help partly |
|  |  | 0 points: Need great help or rely entirely on others |
| **B.1.11**  **Total points of activities of daily living** | □ | The sum of scores for the above 10 items |
| **B.1**  **Classification of activities of daily living** | □ | Unimpaired: 100 points  Mildly impaired: 65-95 points  Moderately impaired: 45-60 points  Severely impaired: ≤40 points |

**B.2 Assessment of mental status**

| **B.2.1**  **Cognitive function** | Test | “I say three things, please repeat them, and remember, I'll ask you later”: apple, watch, flag |
| --- | --- | --- |
|  |  | (1) Clock drawing test: “Please draw a circular clock here and mark the clock at 10:45” |
|  |  | (2) Recall: “Now tell me, what are the three things I asked you to remember？”  Answer: _______、________、________(Doesn't have to be in order) |
|  | □ | 0 points: Draw the clock right(Draw a closed circle with the pointer in the correct position) and can recall 2-3 words |
|  |  | 1 point: Draw the clock falsely or recall 0-1 word only |
|  |  | 2 points: Cognitive impairment has been identified, such as Alzheimer disease |
| **B.2.2**  **Aggressive behaviour** | □ | 0 points: No physical aggression or verbal aggression |
|  |  | 1 point: There are several physical attacks per month or several verbal attacks per week |
|  |  | 2 points: There are several physical attacks per week or verbal attacks per day |
| **B.2.3**  **Depression symptoms** | □ | 0 points: None |
|  |  | 1 point: Depressed, silent, unwashed and inactive |
|  |  | 2 points: Suicidal thoughts or behaviors existed |
| **B.2.4**  **Total points of mental status** | □ | The sum of scores for the above 3 items |
| **B.2 Classification of mental status** | □ | Unimpaired: 0 points  Mildly impaired: 1 point  Moderately impaired: 2-3 points  Severely impaired: 4-6 points |

**B.3 Assessment of sensory and communication**

| **B.3.1**  **Consciousness level** | □ | 0 points: Be conscious and alert to the surroundings |
| --- | --- | --- |
|  |  | 1 point: Hypersomnia, characterized by excessive sleep duration. Wake up when calling or pushing your limbs, and be able to talk or execute instructions correctly. Once stop the stimulation you then continue to fall asleep |
|  |  | 2 points: Lethargy, general external stimulation does not enable you to awaken: When given a strong stimulus, you can have a short period of conscious awareness and can answer questions briefly. When stimulation weakens, you will fall asleep quickly |
|  |  | 3 points: Coma, when in a slight coma, you can avoid pain stimulation with a painful expression; when in a deep coma you are no response to the stimulus(If coma is assessed, severe disability is directly assessed and the following items may not be assessed) |
| **B.3.2**  **Vision (wearing glasses)** | □ | 0 points: Can see the standard font on the books or newspapers clearly |
|  |  | 1 point: Can see big fonts clearly: but cannot see the standard font on the books or newspapers clearly |
|  |  | 2 points: Limited vision, unable to see the newspaper headlines clearly, but can discern objects |
|  |  | 3 points: It is difficult to discern an object, but the eyes can follow the object and only see light, color and shape |
|  |  | 4 points: Without vision, the eye cannot follow an object |
| **B.3.3**  **Hearing** | □ | 0 points: Can talk normally, can hear the sound of TV, telephone, doorbell |
|  |  | 1 point: Cannot hear clearly when speaking softly or more than 2 meters away |
|  |  | 2 points: It is difficult to communicate normally. It is necessary to speak loudly or in a quiet environment. |
|  |  | 3 points: Only when the speaker speaks loudly or slowly can you hear part of it |
|  |  | 4 points: Cannot hear |
| **B.3.4**  **Communication** | □ | 0 points: Can communicate with others without any difficulties |
|  |  | 1 point: Can express your needs and understand what others are saying, but need more time or help |
|  |  | 2 points: It is difficult to express your needs and understand other's words |
|  |  | 3 points: Cannot express your needs and understand other's words |
| **B.3 Classification of sensory and communication** | □ | Unimpaired: Conscious, with vision and hearing is rated as 0 points or 1 point and communication is rated as 0 points  Mildly impaired: conscious, But vision or hearing is rated as 2 points, or communication is rated as 1 point  Moderately impaired: Conscious, but at least one item in vision or hearing is rated as 3 points, or communication is rated as 2 points. Or hypersomnia, vision or hearing is rated as 3 points and below, and communication is rated as 2 points and below  Severely impaired: Conscious or hypersomnia, but at least one of vision or hearing is rated as 4 points: or communication is rated as 3 points. Or lethargy/coma |

**B.4 Assessment of social participation**

| **B.4.1**  **Living** | □ | 0 points: In addition to taking care of your personal life (such as eating, washing, and dressing), you can do housework (Such as cooking, laundry) or manage household affairs |
| --- | --- | --- |
|  |  | 1 point: In addition to taking care of your personal life, you can do housework but not satisfactory, and household affairs arrangements are not well organized |
|  |  | 2 points: You can take care of your personal life; can do housework with other's help but not satisfactory |
|  |  | 3 points: Can take care of your basic life affairs (such as eating, stool and urine). Can wash and gargle under supervision |
|  |  | 4 points: Personal basic life affairs (such as diet and stool and urine) require partial or total dependence on others |
| **B.4.2**  **Working** | □ | 0 points: Originally skilled mental or physical work can be done as usual |
|  |  | 1 point: Decrease in working ability about originally skilled mental or physical work |
|  |  | 2 points: Decrease obviously in working ability about originally skilled mental or physical work, and partly skills have been forgotten |
|  |  | 3 points: Only a few fragments of skilled work be retained and skills have been forgotten |
|  |  | 4 points: All previous knowledge or skills disappear |
| **B.4.3**  **Time/space orientation** | □ | 0 points: The time concept (year, month, day, hour) is clear; you can go far alone and quickly grasp the position of the new environment |
|  |  | 1 point: The time concept is declining, you can know the year, month, and day clearly, but sometimes confuse for a few days; you can go to the near street, and know the name and location of the current residence, but do not know the home route |
|  |  | 2 points: The time concept is poor, you cannot know the year, month, and day clearly, may know the first half or the second half of the year. Can only act alone near home, know only the name of the place of residence, do not know the location |
|  |  | 3 points: The time concept is very poor, you cannot know the year, month, and day, may know the morning or afternoon. Can only go to the neighbor's home to play, do not know the name and location of the place of residence |
|  |  | 4 points: No time concept; can't go out alone |
| **B.4.4**  **Distinguish persons** | □ | 0 points: Know the relationship of people around you. Know the meaning of terms like uncles, grandparents, aunts, nephews and nieces. Can distinguish the general age and identity of strangers, and can call them appropriately |
|  |  | 1 point: Can identify the family relationship only. Cannot distinguish the general age of strangers, and cannot call them appropriately |
|  |  | 2 points: Can call family members only, or can only call follow others, but do not know their relations |
|  |  | 3 points: Know family members who often live with you only, can call children or grandchildren, can distinguish acquaintances and strangers |
|  |  | 4 points: Know only the protectors, cannot distinguish acquaintances and strangers |
| **B.4.5**  **Social communication** | □ | 0 points: Participate in society, have certain adaptability in the social environment, treat people appropriately |
|  |  | 1 point: Being able to adapt to a simple environment and actively in contact with people. It is difficult for others to find your intelligence problems when meeting people for the first time. Cannot understand metaphor |
|  |  | 2 points: Out of society, can be passive contact, cannot take the initiative to treat people, have many uncomfortable words in the conversation. Easy to be cheated |
|  |  | 3 points: Can barely communicate with others, the content of the conversation is unclear and the expression is inappropriate |
|  |  | 4 points: Difficult to make contact with others |
| **B.4.6**  **Total points of social participation** | □ | The sum of scores for the above 5 items |
| **B.4 Classification of social participation** | □ | Unimpaired: 0-2 points  Mildly impaired: 3-7 points  Moderately impaired: 8-13 points  Severely impaired: 14-20 points |
