# Supplementary material for: A Multidimensional Assessment of Activities of Daily Living, Mental Status, Communication, and Social Abilities Among Older Adults in Shenzhen, China: Cross-Sectional Study
Source: JMIR Public Health Surveill. 2023 Aug 10;9:e43612. doi: 10.2196/43612 (PMC10450528; doi:10.2196/43612)
Supplement: Multimedia Appendix 3 [file publichealth_v9i1e43612_app3.doc]

Table S1. Results of the *P* values for the difference in the proportion of overall ability impairment compared pairwise between different age groups.^a^

| Age group (years) | 60-64 | 65-69 | 70-74 | 75-79 | 80-84 | 85-89 |
| --- | --- | --- | --- | --- | --- | --- |
| 65-69 | <.001 | N/A^b^ | N/A | N/A | N/A | N/A |
| 70-74 | <.001 | <.001 | N/A | N/A | N/A | N/A |
| 75-79 | <.001 | <.001 | <.001 | N/A | N/A | N/A |
| 80-84 | <.001 | <.001 | <.001 | <.001 | N/A | N/A |
| 85-89 | <.001 | <.001 | <.001 | <.001 | <.001 | N/A |
| ≥90 | <.001 | <.001 | <.001 | <.001 | <.001 | <.001 |

^a^: Pairwise comparisons were performed using the proportion test. The statistical significance was set at *P*<.05.

^b^N/A: not applicable.

Table S2. Results of the *P* values for the difference in the proportion of overall ability impairment compared pairwise between different education groups.a

| Education | Illiterate or semi-illiterate | Primary or junior high school | Senior high or technical school | College degree or above |
| --- | --- | --- | --- | --- |
| Primary or junior high school | <.001 | N/A^b^ | N/A | N/A |
| Senior high or technical school | <.001 | <.001 | N/A | N/A |
| College degree or above | <.001 | <.001 | 1.00 | N/A |
| Missing | <.001 | 1.00 | <.001 | <.001 |

^a^: Pairwise comparisons were performed using the proportion test. The statistical significance was set at *P*<.05.

^b^N/A: not applicable.

Table S3. Results of the *P* values for the difference in the proportion of overall ability impairment compared pairwise between different marital status groups.a

| Marital status | Never married | Currently married | Divorced or widowed |
| --- | --- | --- | --- |
| Currently married | .02 | N/A^b^ | N/A |
| Divorced or widowed | 1.00 | <.001 | N/A |
| Missing | .81 | <.001 | <.001 |

^a^: Pairwise comparisons were performed using the proportion test. The statistical significance was set at *P*<.05.

^b^N/A: not applicable.

Table S4. Results of the *P* values for the difference in the proportion of overall ability impairment compared pairwise between different living situation groups.a

| Living situation | Live alone | Live with spouse | Live with children | Live with other relatives | Live with nonrelatives | Nursing home |
| --- | --- | --- | --- | --- | --- | --- |
| Live with spouse | <.001 | N/A^b^ | N/A | N/A | N/A | N/A |
| Live with children | <.001 | 1.00 | N/A | N/A | N/A | N/A |
| Live with other relatives | .49 | .50 | .30 | N/A | N/A | N/A |
| Live with nonrelatives | <.001 | <.001 | <.001 | <.001 | N/A | N/A |
| Nursing home | 1.00 | .01 | .007 | 1.00 | 1.00 | N/A |
| Other means and missing | <.001 | <.001 | <.001 | 1.00 | <.001 | .42 |

^a^: Pairwise comparisons were performed using the proportion test. The statistical significance was set at *P*<.05.

^b^N/A: not applicable.
